# Atypical cutaneous manifestation of Erdheim-Chester disease successfully treated using medical and laser therapy

**DOI:** 10.1016/j.jdcr.2025.06.039

**Published:** 2025-07-12

**Authors:** Hasina Maredia, Ronald S. Go, Hafsa M. Cantwell, Julio C. Sartori-Valinotti

**Affiliations:** a Department of Dermatology, Mayo Clinic, Rochester, Minnesota; b Department of Hematology and Oncology, Mayo Clinic, Rochester, Minnesota; c Center for Aesthetic Medicine and Surgery, Mayo Clinic, Rochester, Minnesota

**Keywords:** complex medical dermatology, Erdheim-Chester disease, hematology, nonablative fractional resurfacing laser, pulsed-dye laser, quality of life

## Introduction

Erdheim-Chester disease (ECD) is a rare non-Langerhans histiocytic disorder, primarily affecting long bones and visceral organs. It tends to occur in the fifth to seventh decades of life with a slight male predominance.^1^ Cutaneous manifestations occur in only 25% of ECD patients, primarily as xanthomatous, red-brown papules on the face, neck, axillae, and/or groin.^1^, ^2^, ^3^ Because of the rarity of ECD, diagnosis and treatment can often be delayed and require a multispecialty approach. In this report, we raise awareness of an unusual cutaneous manifestation of ECD and demonstrate successful treatment with a multimodal approach of topical, systemic, and laser treatments.

## Case report

**Fig 1:**
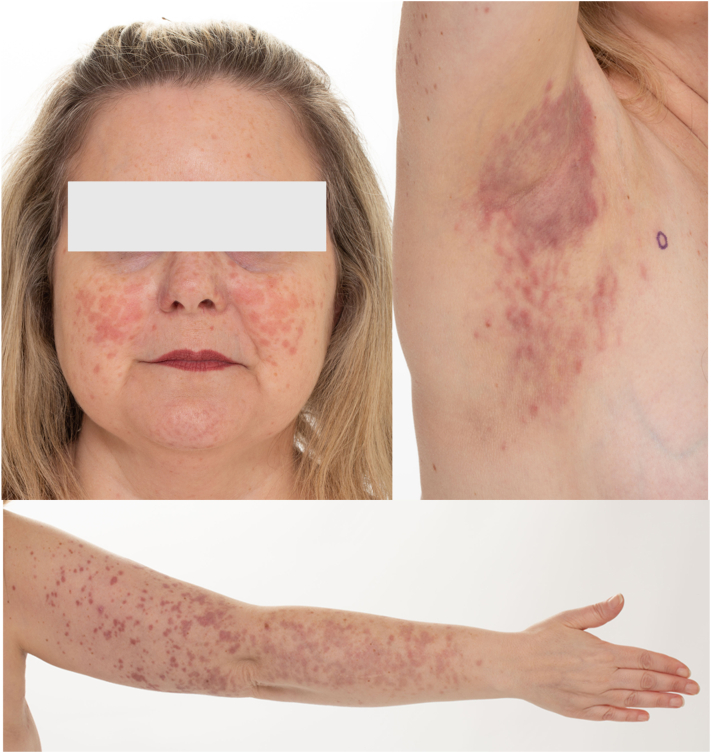
Erdheim-Chester disease presented as erythematous pink-purple papules that coalesced into pruritic tender plaques over the face, trunk, and extremities.

This retrospective case report evaluated a 44-year-old female with Fitzpatrick skin type II who presented with a 6-month history of erythematous papules that coalesced into pruritic, tender plaques over the face, followed by similar lesions over the trunk and extremities (Fig 1). Four months later, she developed lower extremity pain, polydipsia, and polyuria. She did not have any fevers, night sweats, or weight loss. She had 2 separate skin biopsies performed. Both specimens showed that the histiocytes stained positive for CD163, CD68, factor 13A, CD11c (partial), and S100 (focal) and negative for CD1a and *BRAFV600E.* Both specimens underwent next-generation sequencing to analyze DNA and RNA twice (Tempus xT gene panel). DNA and RNA sequencing did not reveal any pathogenic mutation or fusion, including *BRAF* or other activating gene variants in the mitogen-activated protein kinase-extracellular signal-regulated kinase pathway. Magnetic resonance imaging showed a pituitary microadenoma, and nuclear bone scan showed bilateral lower extremity uptake. Computed tomography-positron emission tomography showed osteosclerotic lesions in the bones, with mild-to-moderate increased uptake in the bilateral proximal and distal femoral metaphyses and tibias. She was diagnosed with ECD. Additional workup revealed no cardiac involvement on echocardiogram, no cytopenia, and bone marrow biopsy negative for concomitant myeloid disorder.

**Fig 2:**
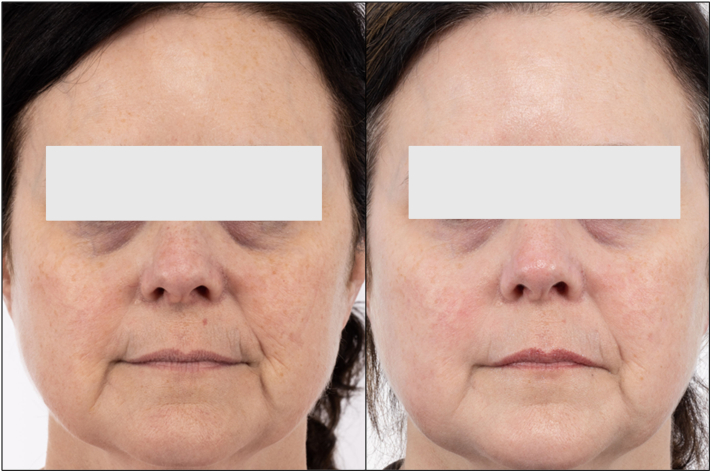
(*Left*) The patient was treated initially with cobimetinib, with improvement in the erythematous macules after completing 2 years of treatment. (*Right*) For the remaining erythema, dyschromia, and rhytides, she was treated with 595 nm pulsed-dye laser once then thulium 1927 nm (Fraxel) handpiece twice with improvement.

Cobimetinib 40 mg daily was initiated with resolution of her systemic symptoms and a negative positron emission tomography scan by 6 months. There was a 70% improvement of her cutaneous findings, with flattening of the plaques but persistent brown postinflammatory hyperpigmentation. After 2 years on cobimetinib, she had systemic remission of disease but developed medication side effects, including cognitive changes, joint pain, diarrhea, and fatigue. Cobimetinib was discontinued. She was transitioned to topical tacrolimus applied to areas with residual cutaneous involvement. She had remaining facial hyperpigmentation, persistent erythema and telangiectasias, and prominent rhytids that developed in the setting of ECD and treatment sequelae, impacting her sense of self and quality of life. The hyperpigmentation was initially treated with compounded 12% hydroquinone, 5% vitamin C, and 6% kojic acid. For persistent erythema, her face was treated with 1 session of pulsed-dye laser (PDL) (Vbeam Perfecta, Candela Laser). The PDL consisted of a 595 nm wavelength, and a 7 mm spot size was used. For the telangiectasias on the nose, fluence of 14 J/cm^2^ and 40 ms pulse duration were used, and for the bilateral cheeks and chin, fluence of 9 J/cm^2^ and 6 ms pulse duration were used. She subsequently underwent 2 sessions of nonablative fractional resurfacing laser (Fraxel Dual 1550/1927 Laser System, Solta) using the thulium 1927 nm handpiece with treatment level 4, 10 J/cm^2^ fluence, and 8 passes. She achieved 95% improvement on her face to her satisfaction (Fig 2).

## Discussion

Our case report demonstrated the importance of recognizing atypical cutaneous manifestations of ECD, beyond the typical xanthelasma-like findings, and the value of multimodal treatment approaches. In this case, the patient presented with cutaneous findings of erythematous papules and plaques over the face as well as the trunk, which has not been a commonly reported feature in the literature. Typical findings are xanthelasma-like periorbital lesions or yellow-brown papulonodules and plaques on the face, neck, and skin folds.^1^, ^2^, ^3^ The rarity and nonspecific systemic symptoms of ECD can lead to a delayed diagnosis, making it important to raise awareness among dermatologists of the wide range of cutaneous presentations of ECD.

For treatment, this case supports the effectiveness of cobimetinib for *BRAFV600E-*negative ECD in combination with a multimodal approach with topical and laser therapy.^3^^,^^4^ Cobimetinib is an oral medication that inhibits mitogen-activated protein kinase regulating cell growth.^4^ Side effects, including gastrointestinal symptoms, fatigue, and musculoskeletal issues, can limit long-term continuation of it, as occurred with our patient. Because of a probable combination of ECD and sequelae of prednisone and cobimetinib, our patient had accelerated aging with the development of rhytides, facial erythema, textural changes, and hyperpigmentation. Laser treatments are increasingly incorporated into the management of medical skin diseases and their sequelae, including rosacea, melasma, and connective tissue diseases.^5^ PDL is used frequently in dermatology with broad applicability, targeting the red chromophore seen in vascular conditions. Prominent telangiectasias and erythematous macules are a rare manifestation of ECD, but this can be seen in a variety of dermatological conditions, such as scleroderma, as well as secondary to treatments like prednisone. PDL was well-tolerated and provided positive results in treating postinflammatory macules in this case. It is furthermore a versatile laser that can be used in combination with other light-based devices. Thulium 1927 nm was used following PDL to significantly improve other cutaneous sequelae of hyperpigmentation and rhytids to her satisfaction and was also well tolerated without adverse events. For more classic ECD periorbital xanthomatous lesions, carbon dioxide laser has also been utilized successfully in the literature.^5^

There are limited data on the use of light-based devices for cases with complex medical and/or dermatological conditions, but it has the potential to greatly improve the quality of life of patients who are reminded of their disease from visible changes on their skin. This case demonstrates that light-based devices combined with systemic and topical treatment can meaningfully improve skin manifestations and sequelae of ECD. As such, additional investigation of the safety and efficacy of light-based devices among other complex dermatological conditions is warranted in a wider-scale prospective study.

## Conflicts of interest

None disclosed.
